# Variable Presentations of Sclerosing Pneumocytoma: Two Cases Highlighting Divergent Fluorodeoxyglucose Metabolism and Carcinoid Tumorlet Association

**DOI:** 10.7759/cureus.83140

**Published:** 2025-04-28

**Authors:** Alexander N Weisberger, Jesse Liou, Hadi Shojaei, Suad Taraif, David D Shersher, Wissam Abouzgheib

**Affiliations:** 1 Division of Pulmonary Medicine, Cooper Medical School of Rowan University, Camden, USA; 2 Division of Pulmonary Medicine, Cooper University Hospital, Camden, USA; 3 Department of Pathology, Cooper University Hospital, Camden, USA; 4 Department of Thoracic Surgery, Cooper University Hospital, Camden, USA

**Keywords:** 18f-fdg, benign lung tumors, carcinoid tumorlet, pulmonary sclerosing pneumocytoma, robotic bronchoscopy

## Abstract

Pulmonary sclerosing pneumocytomas are rare benign tumors, with some cases demonstrating potential for aggressive metastasis. Patients typically are asymptomatic with a nodule/mass seen on incidental imaging; however, they can present with nonspecific symptoms such as cough, shortness of breath, and chest pain. Diagnosis can only be confirmed with biopsy, and computed tomography-guided or fluoroscopy-guided needle biopsies are routinely used. Yet, with the advancement of technology and increased physician proficiency, robotic bronchoscopy can be safely and effectively used to obtain a tissue diagnosis. In this report, we present two cases of sclerosing pneumocytoma tumors. The first case was an incidentally encountered tumor that was found to be hypermetabolic on positron emission tomography (PET) imaging. It was diagnosed using robotic bronchoscopy and resected via lobectomy. The second case was an incidentally encountered tumor that was hypometabolic on PET imaging. It was diagnosed after wedge resection, which also revealed an adjacent carcinoid tumorlet.

## Introduction

Sclerosing pneumocytomas (SPs) are rare, benign pulmonary neoplasms that have a predilection toward non-smoking females in the fifth decade of life [1]. The presentation is usually asymptomatic, with most tumors being discovered on routine chest radiograph [2]. With respect to positron emission tomography (PET) imaging, these tumors can have mixed uptake levels of 18F fluorodeoxyglucose (FDG), with most tumors exhibiting low to moderate standardized uptake value (SUV), while others can be in the range of that of a malignancy, which necessitates biopsy to further determine management [3-5].

The two non-surgical methods of peripheral lung lesion biopsy are bronchoscopic transbronchial biopsy and transthoracic biopsy. Despite having a higher diagnostic yield than bronchoscopy, transthoracic biopsy comes with a higher rate of complications, including pneumothorax and the need for tube thoracostomy. Robotic-assisted bronchoscopy has been shown to have similar diagnostic yield, yet significantly lower rates of complications as compared to transthoracic biopsy of peripheral lung lesions [6,7].

Pulmonary carcinoid tumors are neuroendocrine tumors that make up approximately 2% of all lung tumors. Though often asymptomatic, these tumors can present with pulmonary symptoms such as cough, chest pain, dyspnea, hemoptysis, and wheezing. Rarely, they will cause endocrine symptoms such as flushing and diarrhea via the release of hormones [8]. These tumors are very rarely found growing adjacent to SP tumors, with very few reported in the literature [9].

We present two cases of SP. The first is of a 67-year-old female who presented after the incidental discovery of a lung nodule that was found to have 18F-FDG uptake. Robotic bronchoscopy was used in the biopsy of the nodule, which was finally determined to be a sclerosing pneumocytoma. The second is of a 61-year-old female who presented after a known lung nodule had grown in size over several years, with PET imaging showing the nodule to not have 18F-FDG uptake. It was ultimately diagnosed as a sclerosing pneumocytoma with an adjacent carcinoid tumorlet after surgical resection.

## Case presentation

Case 1 presentation

**Figure 1:**
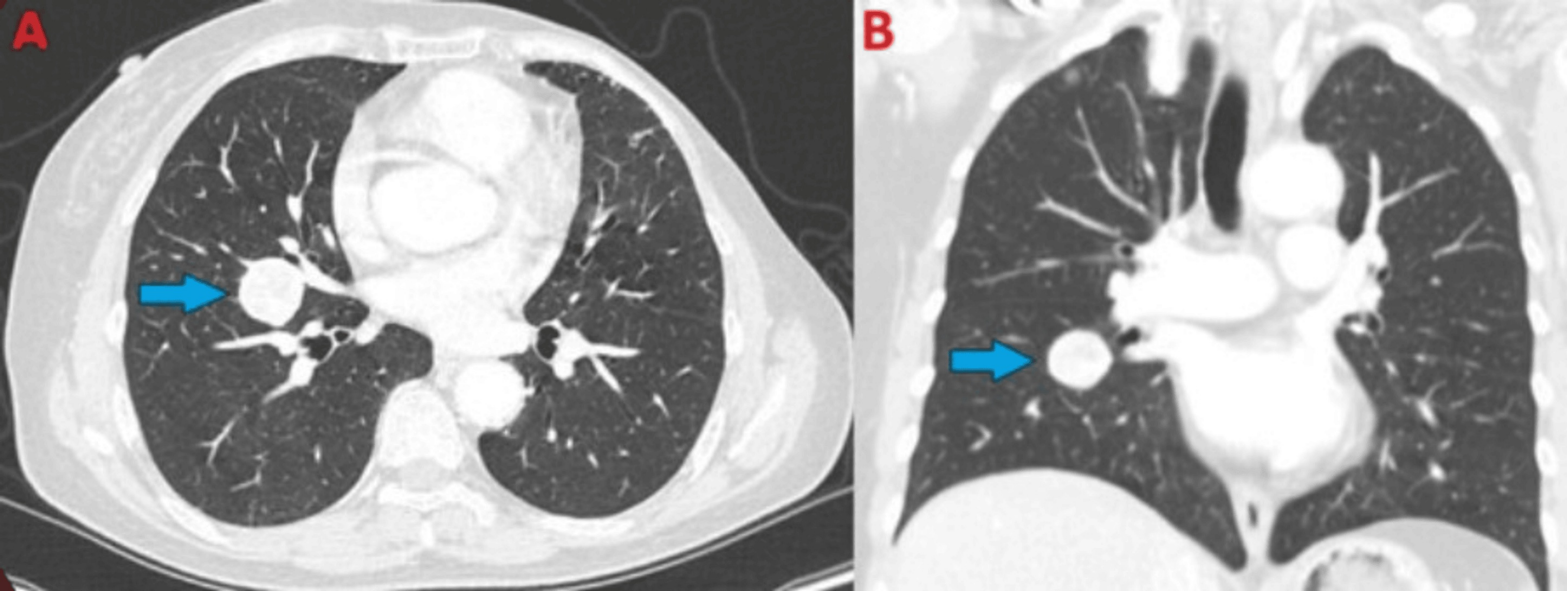
Chest CT (lung windows) demonstrating right circumferential lung nodule (blue arrows) measuring 2.4 x 2.5 cm in axial view (A) and coronal view (B).

**Figure 2:**
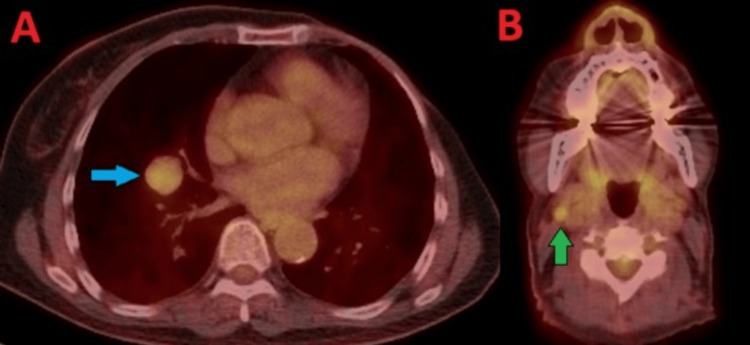
PET scan demonstrating hypermetabolic right-sided lung nodule (A, blue arrow) and hypermetabolic cervical lymph node (B, green arrow).

A 67-year-old female with relevant medical history of treated latent tuberculosis, stage II breast cancer (in remission), and psoriasis presented for pulmonary evaluation after a routine chest radiograph revealed a 2.5 cm rounded right perihilar density. Prior to this, she underwent routine screening for tuberculosis to start a monoclonal antibody (risankizumab) for her psoriasis. The patient was otherwise asymptomatic with normal vitals and physical exam. She had no history of tobacco use. To further characterize the lesion, a computed tomography (CT) was pursued, showing a 2.4 x 2.5 cm solid, circumscribed nodule (Figure 1). Follow-up PET revealed the nodule to be hypermetabolic with a maximum SUV of 3.2 units, along with a hypermetabolic cervical lymph node (Figure 2). There were no mediastinal or hilar lymph nodes that were positive.

**Figure 3:**
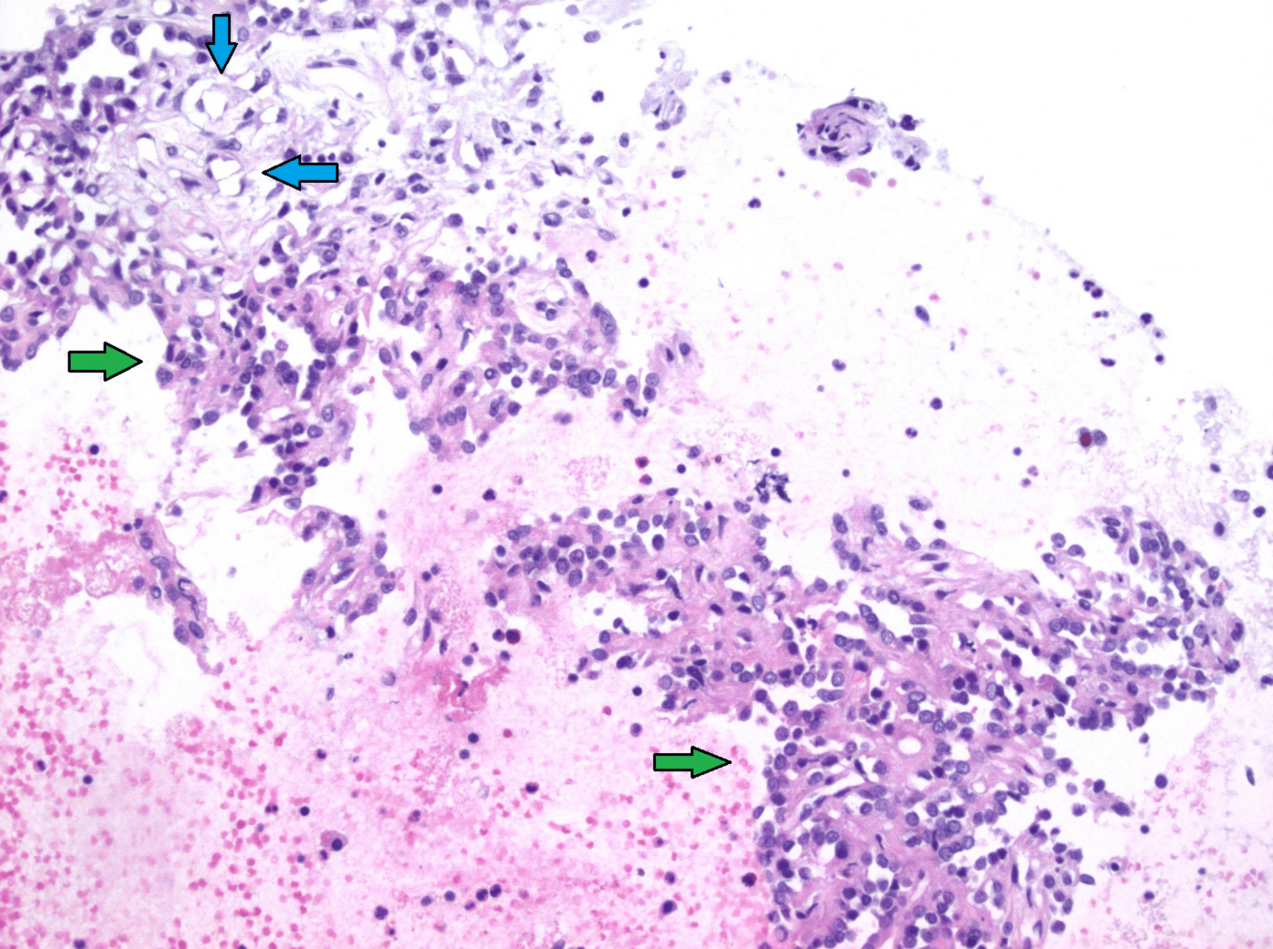
Cell block preparation (hematoxylin & eosin stain, 200x) showing papillary fragments of bland cuboidal tumor cells (green arrows) and adjacent stroma with vacuolated cells (blue arrows).

**Figure 4:**
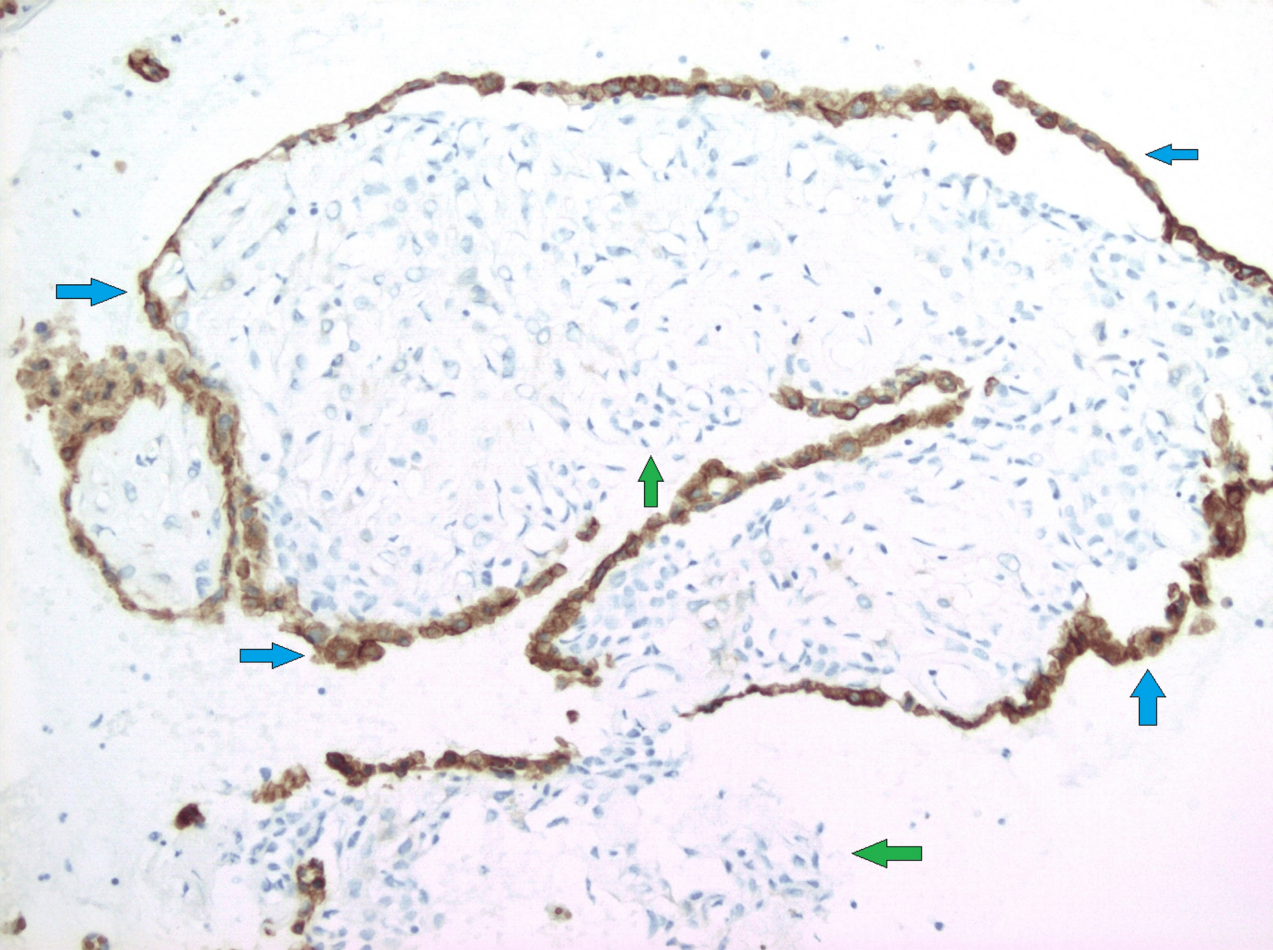
Cell block preparation (AE1/AE3 immunohistochemical stain, 200x) showing surface tumor cells positive for AE1/AE3 (blue arrows), while stromal cells were negative (green arrows).

**Figure 5:**
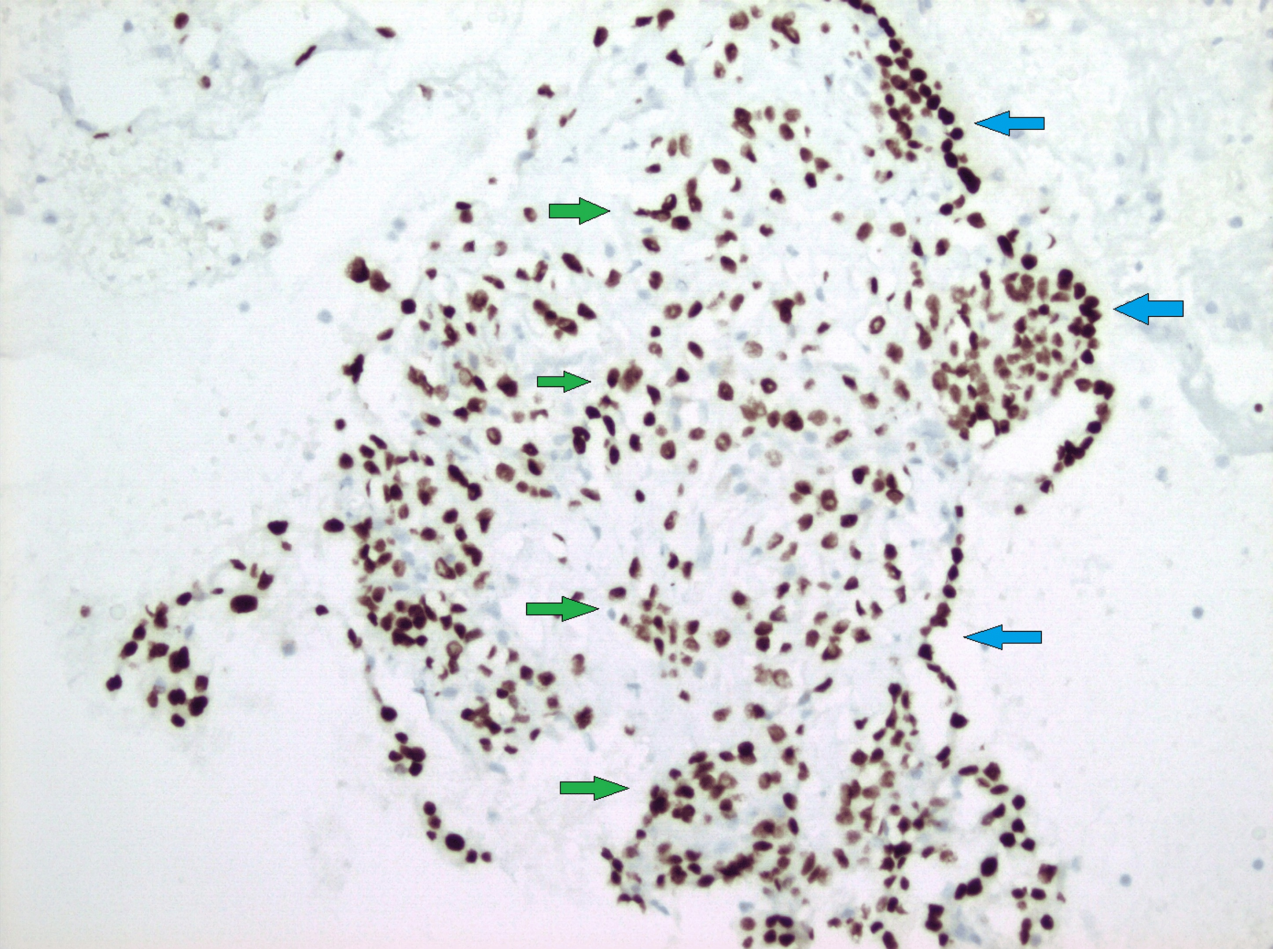
Cell block preparation (TTF-1 immunohistochemical stain, 200x) showing both surface cells (blue arrows) and stromal tumor cells (green arrows) stain positive for TTF-1. TTF-1: thyroid transcription factor 1.

Robotic navigational bronchoscopy was used along with radial endobronchial ultrasound to confirm the location. Transbronchial needle aspiration was used to obtain tissue samples. In addition, no mediastinal or hilar lymphadenopathy was noted on examination with convex endobronchial ultrasound. Cytology revealed papillary fragments lined by monomorphic round to oval cells with bland nuclei, solid nests, and papillary fragments covered by surface cuboidal cells. Immunohistochemical stains showed both stromal cells and surface cells that were positive for thyroid transcription factor 1 (TTF-1) and epithelial membrane antigen (EMA), while negative for GATA-3 and INSM1. A diagnosis of sclerosing pneumocytoma was made based on histomorphology and immunophenotype (Figures 3-5).

**Figure 6:**
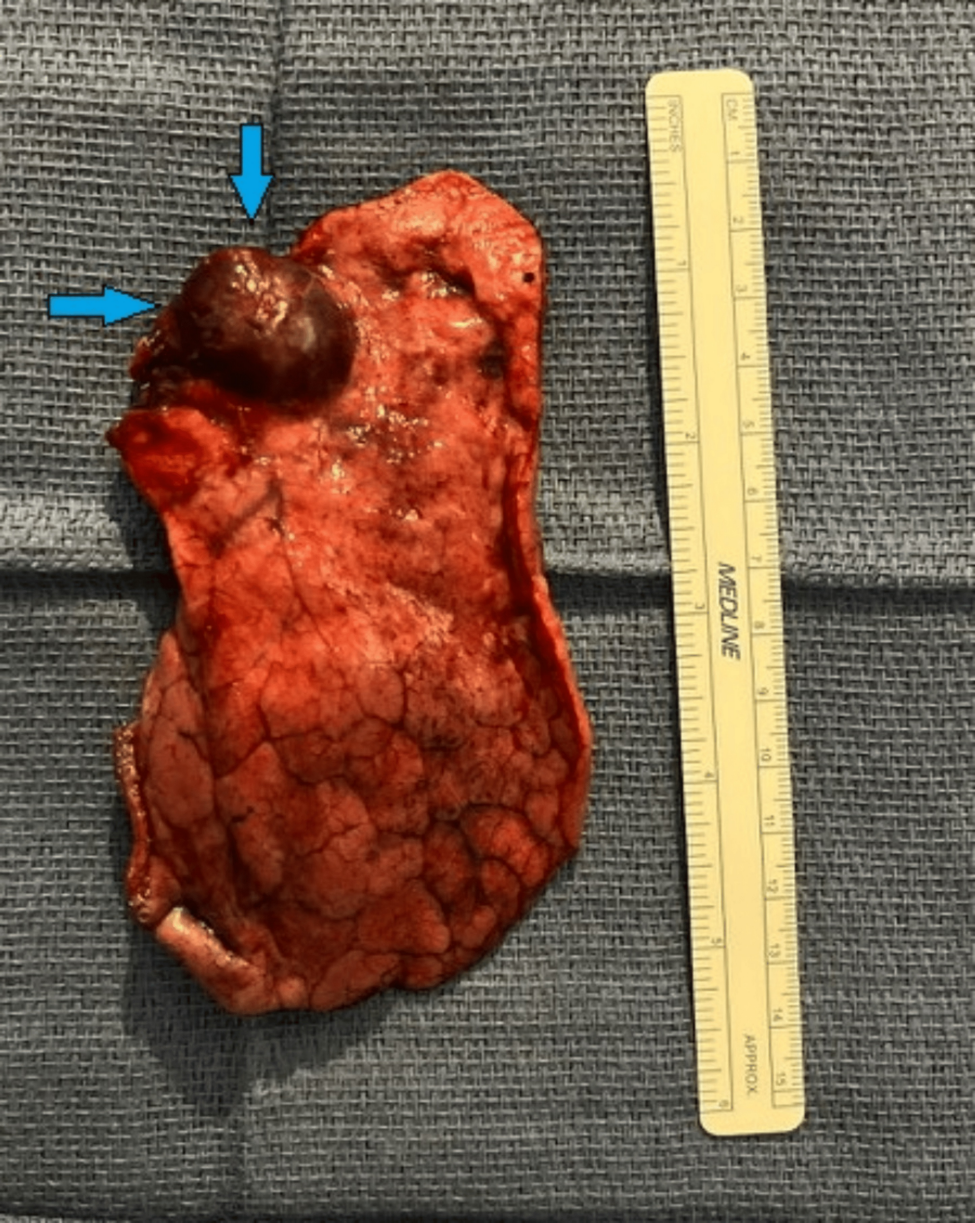
Right middle lobectomy showing sclerosing pneumocytoma located anteriorly along the major fissure (blue arrows).

**Figure 7:**
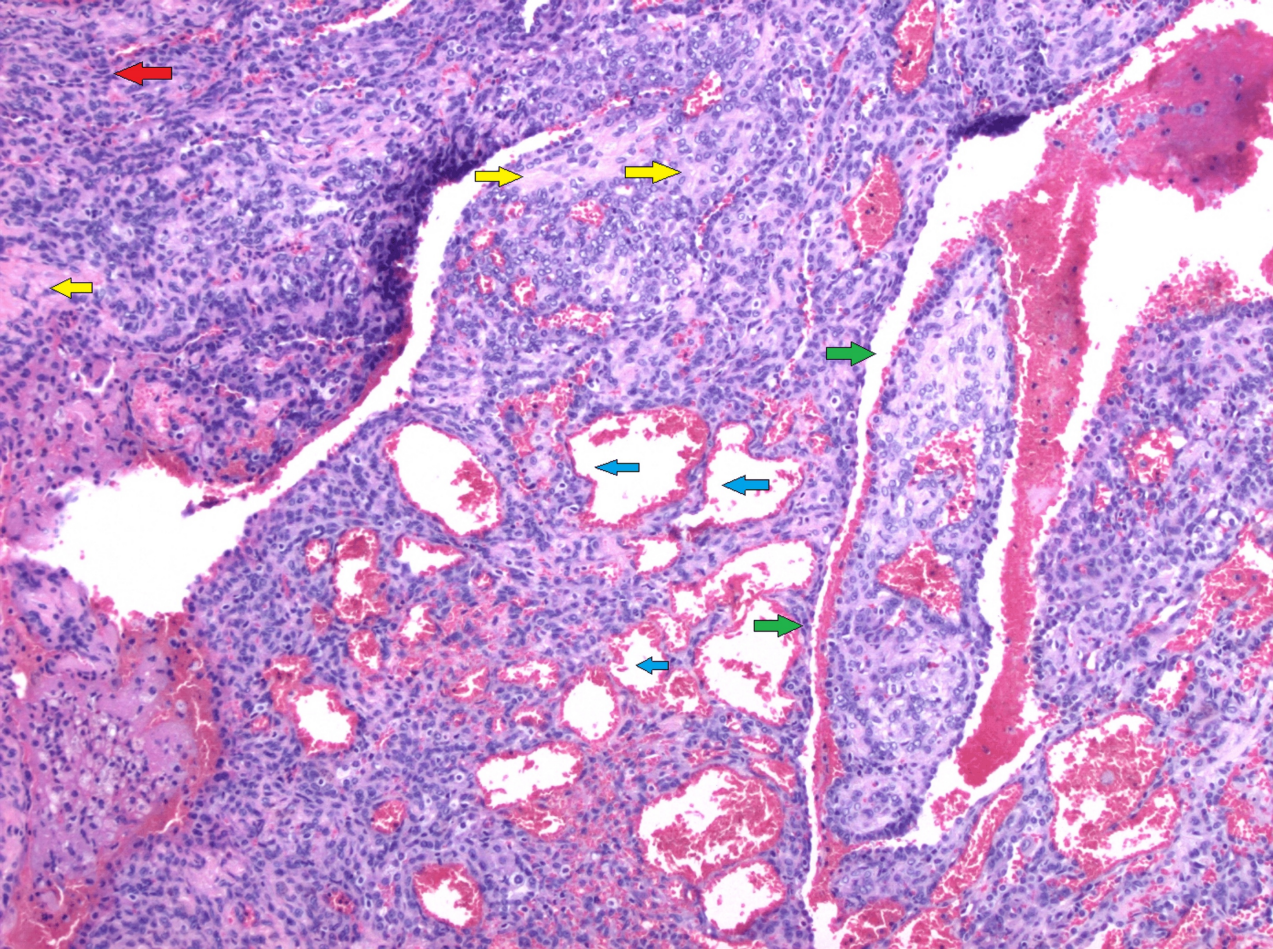
Hematoxylin & eosin (H&E, 100x) stain showing typical variegated tumor architecture with alternating, four different histological patterns: solid (red arrow), papillary (green arrows), hemorrhagic (blue arrows), and sclerotic (yellow arrows) zones.

**Figure 8:**
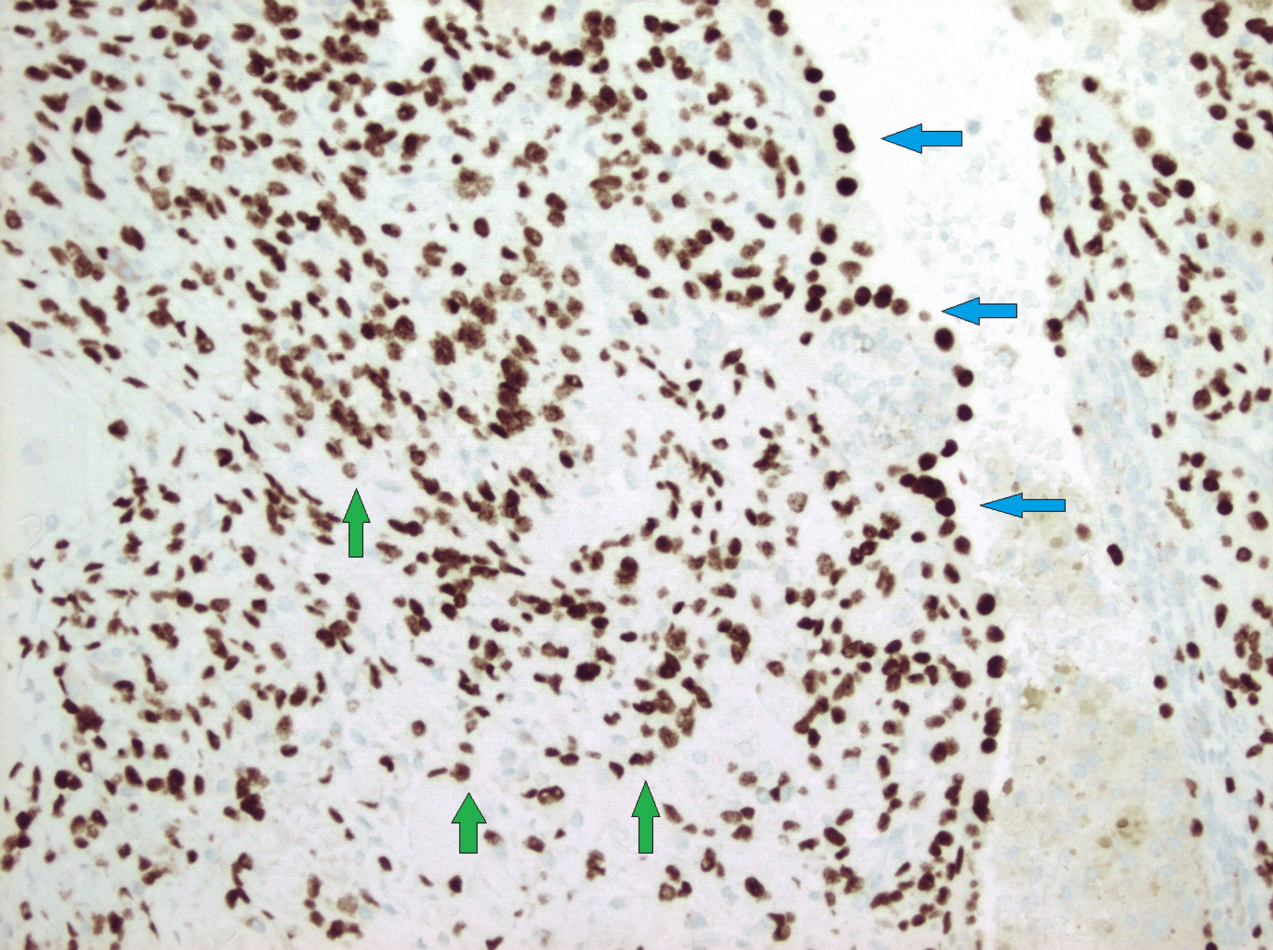
TTF-1 immunohistochemical stain (400x) showing both surface (blue arrows) and stromal tumor cells (green arrows) staining positive for TTF-1. TTF-1: thyroid transcription factor 1.

The patient was referred to thoracic surgery, and a subsequent right middle lobectomy was performed (Figure 6). Notably, the lesion was fused to the right lower lobe anteriorly along the major fissure. There were negative gross margins and no invasion into the lower lobe. Additionally, mediastinal and hilar lymph nodes were sampled, all of which were negative for tumor on subsequent pathological examination. The right middle lobe lung lesion was confirmed to be a sclerosing pneumocytoma with microscopic examination revealing a variegated histologic pattern with hemorrhagic and solid foci alternating with sclerotic zones (Figure 7). There were two cell populations of cuboidal cells lining the papillary foci and a second population of rounded to fusiform stromal cells. Both populations stained positive for TTF-1, but only cuboidal lining cells were reactive to CK7 and CAM 5.2 (Figure 8). EMA was positive in both populations, and GATA-3 and INSM1 were negative. In addition, bronchial, vascular, and parenchymal margins were negative for tumor. Subsequent recovery after the procedure was uneventful.

Case 2 presentation

**Figure 9:**
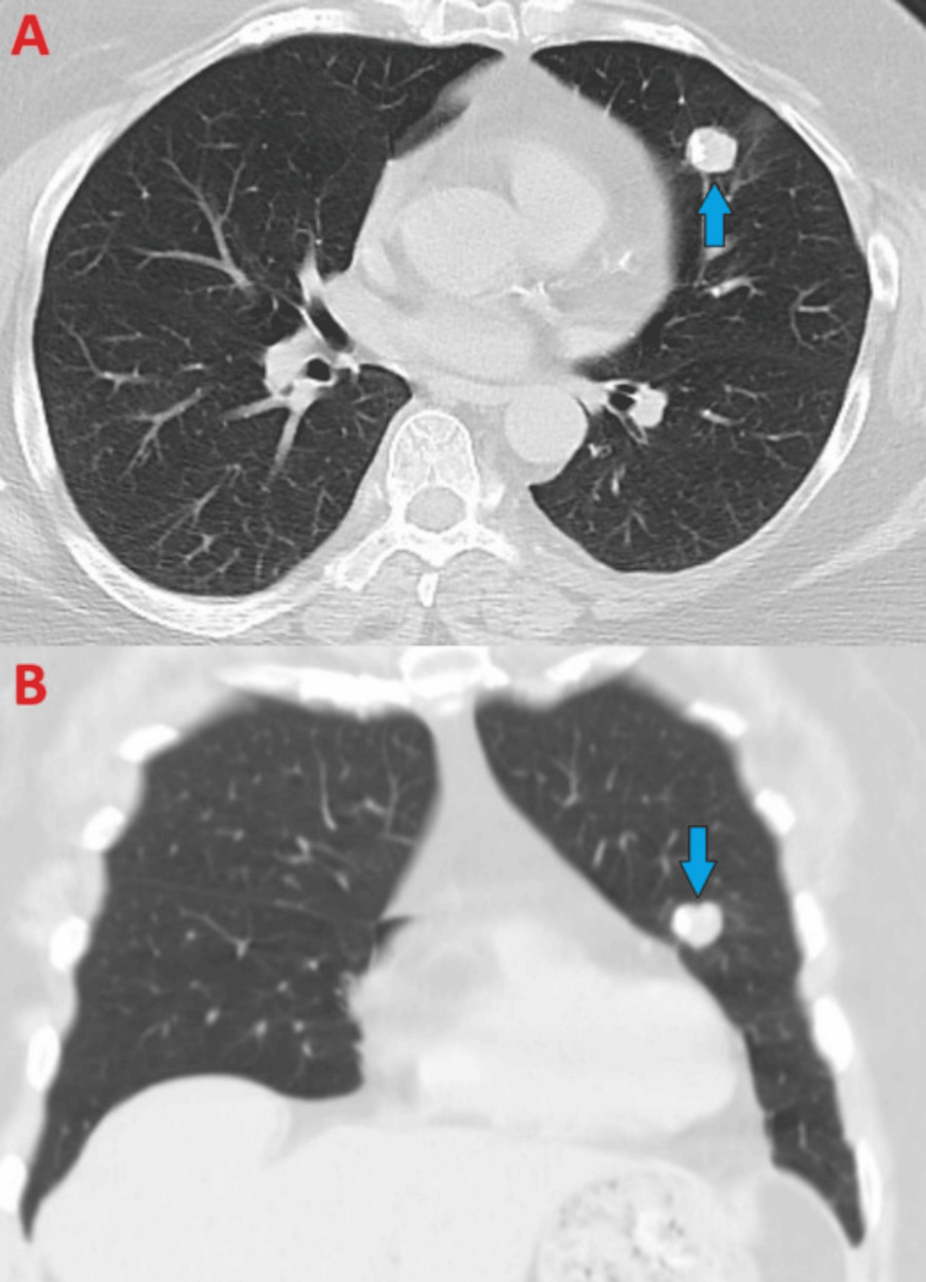
Chest CT (lung windows) demonstrating left circumferential lung nodule with eccentric calcification measuring 1.7 x 1.8 cm in axial view (A, blue arrow) and coronal view (B, blue arrow).

**Figure 10:**
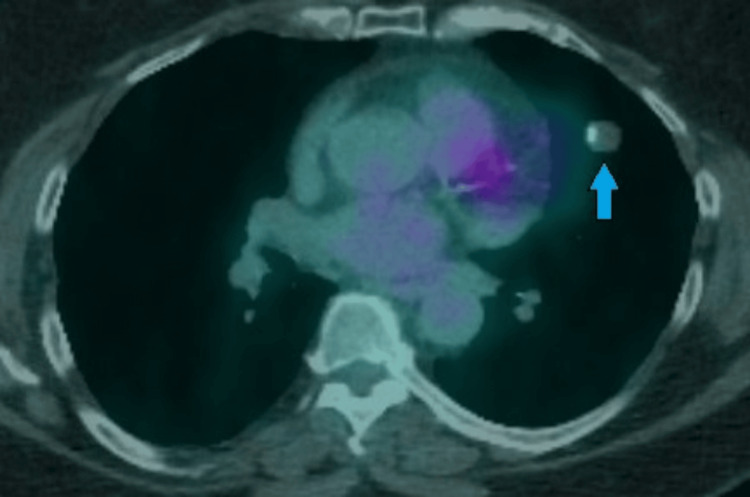
PET scan demonstrating hypometabolic left-sided lung nodule (blue arrow).

A 61-year-old female with no relevant medical history presented to her pulmonologist’s office for evaluation of a left upper lobe nodule that showed interval growth on recent CT. She was otherwise asymptomatic with normal vitals and physical exam. The patient never smoked but was exposed to second-hand smoke growing up. The lesion measured 1.3 cm 12 years prior to presentation. Imaging of the lesion 10 months prior to presentation showed the lesion had grown to 1.7 x 1.8 cm with central calcification, prompting her to pursue workup (Figure 9). Follow-up PET-CT showed the lesion to be non-FDG avid, with no mediastinal lymphadenopathy or hypermetabolic lymph nodes (Figure 10).

It was determined that biopsy would be challenging due to the location of the nodule, so the patient was offered surgical resection by thoracic surgery. Due to patient anxiety, slow growth of the tumor, and patient disinterest in multiple future scans, the decision was made to undergo diagnostic and therapeutic video-assisted thoracoscopic surgical (VATS) wedge resection and mediastinal lymph node dissection.

**Figure 11:**
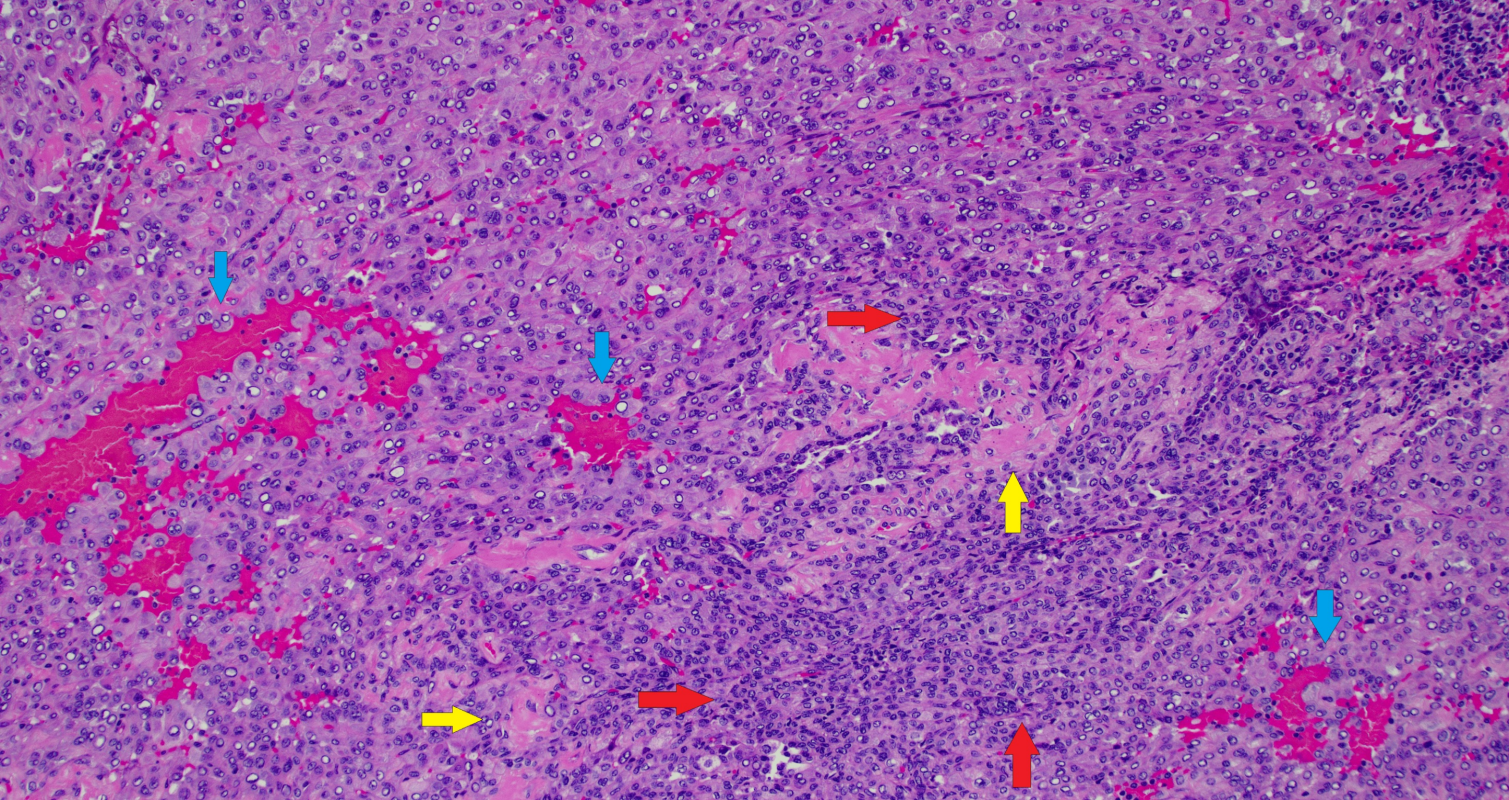
Hematoxylin & eosin (H&E, 100x) stain showing a variegated histologic pattern with predominantly solid growth (red arrows) alternating with sclerotic zones (yellow arrows), as well as focal hemorrhagic areas (blue arrows). There is a dual cell population consisting of epithelioid cells with abundant eosinophilic cytoplasm, prominent intranuclear inclusions, and central nucleoli. Smaller cells with fine chromatin are present within the sclerotic stroma.

**Figure 12:**
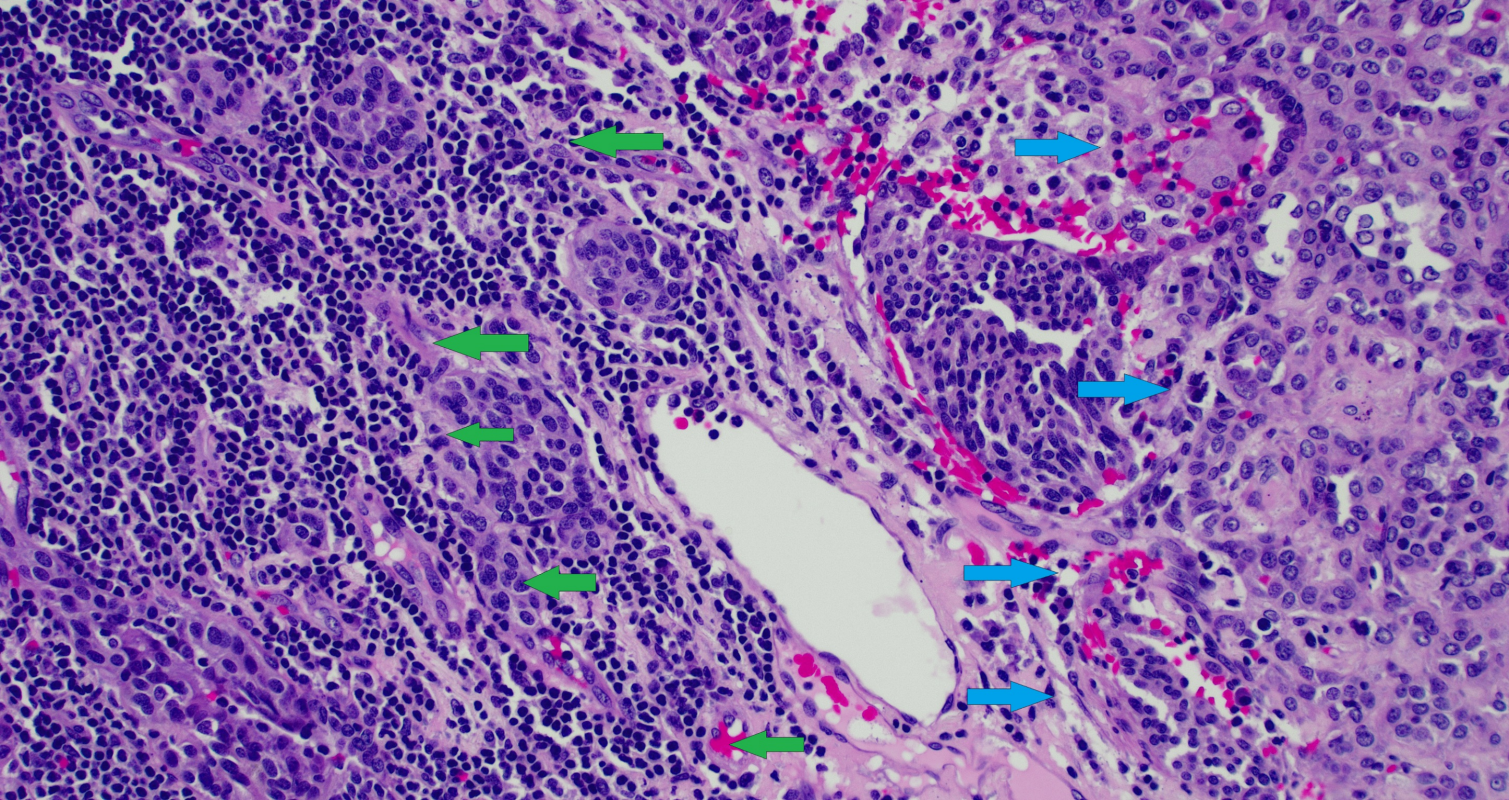
Hematoxylin & eosin (H&E, 200x) stain showing carcinoid tumorlet (green arrows) abutting the sclerosing pneumocytoma (blue arrows).

**Figure 13:**
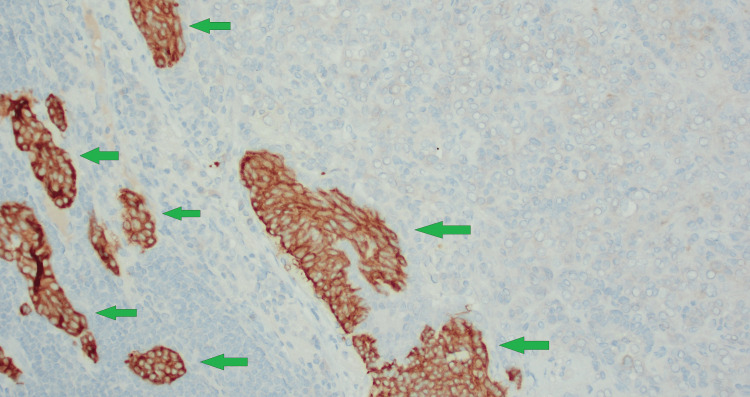
Immunohistochemical analysis (200x) showing carcinoid tumorlet cells staining positive for synaptophysin (green arrows).

At surgery, the lesion was identified and wedge resected with grossly negative margins. An intraoperative pathology consultation confirmed the lesion to be a neoplasm of unclear origin. Mediastinal and hilar lymph nodes were dissected out and sent for pathology. Histological examination showed the lesion to have a variegated histologic pattern with solid, sclerotic, hemorrhagic, and papillary areas. There was a dual-cell population within the tumor, with epithelioid cells with abundant eosinophilic cytoplasm, prominent intranuclear inclusions, central nucleoli, and smaller cells with fine chromatin present within the sclerotic stroma (Figure 11). All cells stained positive for TTF-1 and negative for chromogranin, calcitonin, p40, CD31, CD34, ERG, HMB45, PAX8, and thyroglobulin. There was also a very low proliferation rate on Ki-67 staining. A final diagnosis of sclerosing pneumocytoma was made. All lymph nodes were negative for tumor cells, and a 2 mm carcinoid tumorlet was found abutting the sclerosing pneumocytoma (Figures 12, 13). The parenchymal margin was negative for tumor cells. Subsequent recovery after the procedure was uneventful.

## Discussion

Sclerosing pneumocytoma was first described by Liebow and Hubbell in 1956 as a sclerosing hemangioma [10]. However, further research demonstrated that the tumor is of primitive respiratory epithelial origin rather than vascular, thus prompting a reclassification in 2015 [2,11]. This tumor is more prevalent in female patients, with 83-91.8% of SP patients being female. The age range for incidence is wide, with a range of 23-73 years and a median age of 51 years. It tends to be more prevalent in non-smokers [1,2]. It also tends to be prevalent among patients of East Asian descent [12]. Age, gender, and risk factors were consistent in both of our patients, as the first was a 67-year-old non-smoking female, and the second was a 61-year-old non-smoking female, though the latter patient was exposed to secondhand smoke in her youth.

Radiologically, it typically presents as a single ovoid lesion with a smooth boundary [3]. While these features are less likely to be present in malignancy, the size of the tumor in our first patient raised concern. In addition, most of these tumors have low levels of FDG uptake with SUV < 2.5 units. With an increase in size, some of these tumors demonstrate higher uptake with SUV > 2.5 units, in which malignancy needs to be ruled out [3-5]. Other possibilities include hamartoma, granuloma, and inflammatory myofibroblastic tumor. Biopsy is necessary to guide further treatment and ultimately rule out malignancy. To our knowledge, this would be the second case of SP diagnosed using robotic bronchoscopy [4]. Given the first patient’s history of breast cancer and the presence of lymphadenopathy, there was initial concern that this could be a recurrence. Lymph node sampling was done during the procedure, all of which were negative for neoplastic cells. The slow growth, eccentric calcification, and hypometabolic nature of the second patient’s nodule made malignancy less likely, with hamartoma being the leading differential diagnosis prior to pathology review.

SP is composed of two cell types, i.e., cuboidal surface cells resembling type II pneumocytes and round stromal cells [9], both believed to be of pneumocytic origin. The surface and round cells stain positive for TTF-1 and EMA, confirming their pneumocytic derivation. The tumors contain cells in four possible histologic patterns: papillary, sclerotic, solid, and hemorrhagic [2,13]. In our first patient, the lesion contained all four histologic cell patterns, with hemorrhagic and solid foci alternating with sclerotic zones, while papillary architecture was limited to the periphery. It contained a two-cell population, with cuboidal cells lining the papillary foci and rounded to fusiform stromal cells composing the bulk of the lesion. Both cell populations stained positive for TTF-1 and showed patchy staining for EMA. Our second patient’s tumor also contained all four histologic cell patterns and a two-cell population. All tumor cells stained positive for TTF-1.

The curative treatment for SP is surgical resection. There are multiple surgical techniques that have been performed and have resulted in recurrence-free survival, such as enucleation, wedge resection, and lobectomy [14]. Pneumonectomy and segmentectomy have also been performed. However, careful consideration needs to be taken when making the decision to operate for SP. There is no significant difference in all-cause mortality between SP patients managed with surgery compared to those without surgery. Patients who are symptomatic from the tumor have reported relief of symptoms after surgical resection [15], but again, most patients are asymptomatic [2]. In a study published in 2022, authors reviewed all published cases of SP to date and found that only 1.33% of reported cases were found to be malignant, and only one patient had died due to the disease [15]. Lung resection can lead to serious postoperative adverse events such as prolonged air leak, bronchopleural fistula, pneumonia, acute respiratory failure, hemorrhage, atelectasis, pneumothorax, bronchospasm, pulmonary embolism, and acute respiratory distress syndrome [16]. The risk of not undergoing surgery in biopsy-confirmed SP should be weighed against the risks of surgery, particularly in patients who are at higher risk of postoperative complications. In our first patient, factors that led toward the decision to operate were the hypermetabolic nature of the tumor, size and positioning of the tumor making resection amenable, and the patient’s good physiologic reserve. In our second patient, factors that led toward the decision to operate were patient anxiety, difficulty of biopsy using a less invasive modality, and patient desire not to continue undergoing indefinite surveillance scanning.

Our second patient was found to have an incidental small, 2 mm carcinoid tumorlet after resection on pathology. The incidence of SP growing adjacent to carcinoid tumors is rare, with fewer than 10 cases reported in the literature [9]. The way in which this occurs is unclear, but several hypotheses exist in attempt to explain this phenomenon, including both tumors growing next to each other at the same time, the tumors originating from a common stem cell but differentiating early, and SPs changing their local environment to promote the proliferation of neuroendocrine cells [17].

## Conclusions

SP is a rare benign lung neoplasm that often presents asymptomatically. Despite usually having radiographic features not concerning for malignancy, some tumors will display as hypermetabolic on PET imaging, which drives a need for biopsy. Robotic bronchoscopy can be a useful tool in diagnosing tumors such as these, minimizing risks when compared to CT-guided biopsy or surgical resection. Diagnosis can be confirmed with characteristic histologic architecture and cell types, as well as staining for TTF-1 and EMA. Careful consideration should be taken when considering surgical resection of these tumors. This report adds to the growing body of literature about this rare tumor, particularly having variable 18F-FDG, the ability to be diagnosed with robotic bronchoscopy, and a unique association with carcinoid tumors.
